# Adoption of a COVID-19 Contact Tracing App by Czech Youth: Cross-Cultural Replication Study

**DOI:** 10.2196/45481

**Published:** 2023-11-16

**Authors:** Michal Dolezel, Zdenek Smutny

**Affiliations:** 1 Faculty of Informatics and Statistics Prague University of Economics and Business Prague Czech Republic

**Keywords:** contact tracing, proximity tracing, digital contact tracing, Health Belief Model, technology adoption, COVID-19, qualitative verification, Health Belief Model approach, pandemic crisis, eRouska, eMask

## Abstract

**Background:**

During the worldwide COVID-19 pandemic crisis, the role of digital contact tracing (DCT) intensified. However, the uptake of this technology expectedly differed among age cohorts and national cultures. Various conceptual tools were introduced to strengthen DCT research from a theoretical perspective. However, little has been done to compare theory-supported findings across different cultural contexts and age cohorts.

**Objective:**

Building on the original study conducted in Belgium in April 2020 and theoretically underpinned by the Health Belief Model (HBM), this study attempted to confirm the predictors of DCT adoption in a cultural environment different from the original setting, that is, the Czech Republic. In addition, by using brief qualitative evidence, it aimed to shed light on the possible limitations of the HBM in the examined context and to propose certain extensions of the HBM.

**Methods:**

A Czech version of the original instrument was administered to a convenience sample of young (aged 18-29 y) Czech adults in November 2020. After filtering, 519 valid responses were obtained and included in the quantitative data analysis, which used structural equation modeling and followed the proposed structure of the relationships among the HBM constructs. Furthermore, a qualitative thematic analysis of the free-text answers was conducted to provide additional insights about the model’s validity in the given context.

**Results:**

The proposed measurement model exhibited less optimal fit (root mean square error of approximation=0.065, 90% CI 0.060-0.070) than in the original study (root mean square error of approximation=0.036, 90% CI 0.033-0.039). Nevertheless, perceived benefits and perceived barriers were confirmed as the main, statistically significant predictors of DCT uptake, consistent with the original study (β=.60, *P*<.001 and β=−.39; *P*<.001, respectively). Differently from the original study, self-efficacy was not a significant predictor in the strict statistical sense (β=.12; *P*=.003). In addition, qualitative analysis demonstrated that in the given cohort, perceived barriers was the most frequent theme (166/354, 46.9% of total codes). Under this category, psychological fears and concerns was a subtheme, notably diverging from the original operationalization of the perceived barriers construct. In a similar sense, a role for social influence in DCT uptake processes was suggested by some respondents (12/354, 1.7% of total codes). In summary, the quantitative and qualitative results indicated that the proposed quantitative model seemed to be of limited value in the examined context.

**Conclusions:**

Future studies should focus on reconceptualizing the 2 underperforming constructs (ie, perceived severity and cues to action) by considering the qualitative findings. This study also provided actionable insights for policy makers and app developers to mitigate DCT adoption issues in the event of a future pandemic caused by unknown viral agents.

## Introduction

### Background

During the worldwide COVID-19 pandemic crisis, the role of contact tracing or “the process of identifying, assessing, and managing people who have been exposed to a disease to prevent onward transmission” [1] intensified. This was owing to the fact that an effective treatment was missing and transmission dynamics were high. Along this line, many studies in the field of public administration and policy illustrated the importance of taking rapid and focused action [2,3]. For example, Italian regions with prompt implementation of strict antipandemic measures eventually constructed a more effective contact tracing system for coping with the first pandemic wave than other regions, where containment policies were not that strong [2]. In contrast, comparative studies examining antipandemic measures in different countries have shown that “high levels of strictness in public policy seem to have low effectiveness to stop pandemics similar to COVID-19 driven by mutant viral agents” [4]. Apart from imposing strict measures and stay-at-home instructions, many governments across the globe attempted to unveil the potential of IT.

Digital contact tracing (DCT) is the use of IT to make contact tracing more efficient and effective [5]. In most cases, DCT has been implemented by deploying a specialized mobile app, allowing for data collection through various technological means, such as Bluetooth or location data sharing [6,7]. Accurate and user-friendly DCT can effectively complement strict antipandemic policies, as illustrated by the findings of the initial simulations and follow-up empirical studies [8]. However, in most Western societies, the decision to adopt such an app was left to the citizens [9]. Soon after the introduction of this technology in many countries, the notion of DCT became a subject of heated debates [10]. Although the IT infrastructure and software development was the less problematic issue, many people have turned down the idea of DCT owing to various concerns [11].

The abovementioned situation brought a challenge for the governments and public health authorities [12]. As a matter of fact, DCT requires a high population uptake (56%-95%) to bring the desired effects [13]. It has therefore become important to understand the attitudes and concerns of the general public regarding DCT technologies [14]. Diverse individual motives and attitudes seemed to drive the uptake or refusal of the technology [15]. In that sense, studies probing into these aspects of DCT became a promising tool to explain why many see DCT as a failure or, at minimum, bringing less benefits than was originally hoped for [12]. Although some could hold that the pandemic has ended and it makes little sense to continue broadening the body of knowledge on pandemic-related technology, there are many future research opportunities in this area that should be addressed [16]. Such research findings are needed to help with formulating important postpandemic lessons.

### Prior Work

So far, efforts to map the diverse terrain of DCT from different pragmatic and theoretical perspectives have resulted in a large, steadily growing, and diverse body of knowledge [9,13,17]. Among the first, a survey performed in several European countries and the United States by Altmann et al [18] found a “strong support for the app under both regimes, in all countries, across all subgroups of the population.” Additionally, a high level of willingness was identified from countries such as the United Kingdom [19], Ireland [15], the Netherlands [20], Germany, Switzerland [21], China, the United States [22], and many other countries [23]. In contrast, the initial level of enthusiasm can be contrasted with later reports of skepticism that some studies identified as a saliant position in the public discourse [11]. Interestingly, some recent contributions in a few diverse research communities, including media and communication studies, information systems, human-computer interaction, and human factors, highlighted the possible role of attributes such as altruism (and more broadly prosocial behavior) [24,25] or collectivism [26]. Such factors are often believed to be culturally embedded [27].

Current studies continue to broaden the latter line of thought, for example, by highlighting the role of moral intensity or the extent of a feeling related to moral imperatives [28]. By increasing moral intensity, this can be exploited as an effective driver of influencing people’s decision, for example, whether to adopt a mobile contact tracing app [28]. This is a promising stream of research, as such research endeavors allow for connecting the study of DCT with some other areas of socially receptive medical research, offering adequate conceptual repertoire. The latter stream of research includes examples such as the study of local contextual factors and prosocial motives underpinning voluntary mask wearing [29] or vaccination intentions [30].

### This Replication Study

Through this study, we did not aim to directly contribute to the theory-building efforts of the abovementioned social sciences. Nonetheless, we maintain that it is perhaps a bit early to formulate strong and culture-agnostic conclusions for policy makers in public health IT. More specifically, we argue that mapping the additional pieces of the cultural puzzle related to DCT is highly desirable. As highlighted by Prakash and Das [31], “qualitative studies [focused on DCT adoption] are very few, and more comprehensive studies that combine qualitative and quantitative insights using a mixed-methods approach are not present in the literature.” Broadly stated, people-focused studies are frequently described as context dependent, rendering the role of national culture as one of the foremost factors in this effort [32]. Moray [33], while addressing the human factors community, pointed out the following 2 decades ago: “[t]here are good reasons for believing that the results of ergonomics research in the USA or in Western Europe are not universally applicable.” A similar argument was recently repeated by Jannati [34] regarding DCT in the medical informatics community when highlighting the continuing need for theories underpinning DCT research. Generally, replicating findings in different cultural settings is deemed important to increase credibility and eventually provide generalizability of isolated findings. Together, these aspects contribute to the idea of “cumulative science” [35]. Specifically, in the domain of digital health, national culture is recognized as a salient player in evidence-based interventions, and the need for more cross-national studies was articulated [36,37]. Accordingly, this instrumental replication study represents a step in that direction.

Therefore, in this study, we have reported the results of a cross-cultural, cohort-based replication of the original study by Walrave et al [20]. The aim of this study was to understand how people perceive the role of DCT. At the time of study initiation, this was an innovative and promising technology with a history of implementation and use of approximately 6 months. In the quantitative part, we followed the original study as closely as possible. However, we decided to focus on the cohort of young Czech adults aged 18-29 years, instead of aiming broadly on the population of Czech citizens. Our study was driven by the following research question: *to what extent does the stimuli driving the DCT uptake in the youth population of the Czech Republic differ from those in the population of Belgium?* As the underpinning theory, the original study used the Health Belief Model (HBM) to understand the intentions to adopt DCT. Dating back to 1950s and 1960s, the HBM is a well-established theoretical tool in the domain of social cognition applied to public health problems [38]. In brief, the model was created to “explain preventive health behavior” [39]. Then, using the terminology derived from the HBM, our reasoning associated with this study can be rephrased as follows. We hypothesized that in the former cohort, which lives in a different cultural context than the one described in the original study, the HBM predictors of behavioral intention to adopt a mobile contact tracing app would considerably differ from the original setting in the sense of their distribution.

Such a reasoning was based on 3 foundations. First, differently from Belgium, the Czech Republic introduced a contact tracing app (named eRouska or “eMask”) soon after the COVID-19 pandemic started. Second, the cultural norms and values in Belgium and the Czech Republic, a central European country with a socialist legacy, differ [40]. Among the differences, altruism, a cultural trait described as essential for the success of voluntary contact tracing mechanisms, plays reportedly a weaker role in the Czech society than in some other countries. Third, our cohort of youth (aged 18-29 y) fulfills the characteristics of digital natives, said to include people born from circa 1980s [41,42]. We therefore hypothesized that the adoption of a DCT app would be very natural and obvious for this age cohort, which might have influenced the survey results significantly. In addition, we used the 6D model of national culture by Hofstede et al [43] to compare the cultural traits of both countries. Apart from the quantitative replication of the original study, we have contributed by presenting the qualitative findings of our study, suggesting some extensions of the original HBM constructs.

## Methods

### Study Setting and Context

The Czech Republic is a European country with circa 10 million inhabitants. The inhabitants of the Czech Republic view themselves as belonging neither to the West nor to the East [44]. Historically, the country has a socialist legacy; its predecessor, Czechoslovakia, was a satellite of the Soviet Union from 1948 to 1989. Despite that, the Czech Republic has exhibited significant cultural ties to the German cultural space as long as since the early Middle Age period [45]. With respect to these cultural nuances, the context of our study substantially differed from that of the original study we replicated [40]. In the following sections we have highlighted some factors and events related to the development of the pandemic in the Czech Republic, which are important for understanding the cultural setting.

In March 2020, the Czech government’s reaction to the growing pandemic concerns was rapid. This was based on the close monitoring and evaluation of the pandemic situation in Italy, which many Czech citizens visited for winter holidays before March 2020. On March 12, 2020, the state of emergency, a form of lockdown, was declared. In addition, the Czech Republic was the first European country that declared wearing masks as mandatory from March 19, 2020, onward [46]. In terms of reaction time and level of restrictions, the lockdown can be characterized as a case of “early moderate lockdown,” as termed in the comparative public policy literature [3]. That said, the concrete organizational measures and restrictions differed through time considerably. For example, from March 2020, a formal stay-at-home instruction was legally effective, while containing many exceptions and being enforced by the police only on a case-by-case basis [46].

Practically all measures were loosened before the summer holidays (July to August 2020). Despite the number of steadily growing new cases, it was not until November 2020 when substantial measures were reinstalled. The unwillingness to reintroduce unpopular measures has been interpreted by many as a case of striking populism [47] and attributed to the fact that a regional voting was scheduled for mid-October 2020. The paradoxical aspects of this dramatic shift in governmental strategy for pandemic management were noted globally also, as illustrated by a Cable News Network commentary from October 2020 [48]. Eventually, in March 2021, a strict version of lockdown was introduced, resulting in regular police checks at the limits of 76 Czech counties [46].

Speculatively stated, the abovementioned development comprising inconsistent communication and considerable changes in operational measurements might have led to a significant erosion of the trust in the Czech government over time. Subsequently, many measures, including the latter one, were seen as having debatable impact and were widely criticized by the public. Owing to its dynamics and unpredictability, some scientists and public opinion figures characterized the official communication of the Czech government bluntly as “chaotic, unclear, contradictory and with frequent unexpected twists” [49]. They contrasted it with the considerable level of involvement of both technologists and scientists, highlighting the role of unity and the contribution of do-it-yourself initiatives for handling the pandemic crisis during the initial stage of the pandemic. These facts have been explained in more detail in the *Discussion* section, as we consider the cultural context of strong importance for interpreting the conclusions of this replication.

eRouska, a national contact tracing app, was introduced as a green-field community effort under the wings of the COVID19CZ [50] initiative. This initiative acted as an informal think tank of both practicing technologists and scientists. Apart from eRouska, the think tank conducted several other projects. For example, an effort of Prusa Research to replace the lack of protective shields by using 3D printing has eventually led to a global impact, which stemmed from making the shield designs open source [51]. In addition, under the wings of the same umbrella initiative, biomedical engineering scholars affiliated with a major Czech technical university designed and developed a low-cost ventilator system for emergency use. These illustrations highlight the fact that the public was largely concerned and involved in dealing with the pandemic crisis at its advent. This, unfortunately, seemingly changed through time.

Regarding eRouska, its first version for the Android platform was released on April 11, 2020, and the iPhone Operating System version followed on May 4, 2020 [50]. The app had a simple graphical user interface. Apart from a 1-time SMS campaign, there was no mass media advertising that would promote the adoption of the app among the public. Anecdotal reports associated these missing promotional activities with the cost-saving efforts of the government.

### Sample and Data

We have reported the findings of a population-based, self-reported, and cross-sectional survey with a cohort of young adults aged between 18 and 29 years. The survey was deployed in QuestionPro (QuestionPro Inc [52]), a web survey platform. The survey was available between November 6, 2020, and November 28, 2020 (3 weeks). We recruited study participants by means of convenience sampling. The link to the questionnaire was shared via social network channels (mostly Facebook groups targeted at university students) by posting an advertisement in Czech in these groups. A group of master’s students was involved in the data collection process in return for a course credit, to reach to a more diverse group of young respondents. The respondents were asked to freely share the link to the survey with their personal contacts. Owing to having also an explorative, qualitative component focused on a more broadly defined population, the research project applied no a priori filtering of respondents during data collection. Nonetheless, only the findings related to the target cohort of young adults specified previously have been reported in this paper.

### Ethical Considerations

Given that this was an anonymous survey without a component of social risk (as discussed in guidelines [53]) or personal data collection, no ethical committee approval was sought, as this was not necessary under local regulations [54]. The Prague University of Economics and Business also determined that the study did not require an ethics review. No incentives were offered to respondents.

### Measures of Variables

We closely followed the original study design underpinned by the application of the HBM. The main constructs, namely, perceived susceptibility, perceived severity, perceived benefits, perceived barriers, cues to action, and self-efficacy, were adopted from the original study by Walrave et al [20]. In this study, 5-point Likert-type items, ranging from disagree to agree, were used for all the constructs, except for cues to action, for which a different scale was used (from never to multiple times a day).

Among the HBMs constructs, *perceived susceptibility* was defined as the perceived probability of contracting the COVID-19 infection. This construct was measured using 3 items. An example included, “I am at risk of being infected by the COVID-19 virus.” *Perceived severity* quantified the level of concerns about unwanted consequences when contracting the infection. Again, 3 items were used, for example, “If I were infected by the COVID-19 virus, my health would be severely affected.” *Perceived benefits* were assessed using 6 items, measuring the extent of personal gains when using the app (“...to protect myself from the COVID-19 virus”) or public good (“...I will help public authorities to combat the COVID-19 virus”). The 5 items associated with *cues to action* mapped information consumption regarding the pandemic via different digital channels. These included traditional websites of newspapers, specialized apps, social media channels, messaging apps, email, and newsletters. Measured using 3 items, self-efficacy was defined as the extent of one’s ability to remove constraints and solve problems related to the app. This was either on their own (“I have the knowledge needed...”) or by asking for help (“I can get help from others if I experience difficulties...”). Finally, *behavioral intention* quantified the plan “to use the COVID-19 app” at the present time or in the future.

Owing to the rapid development of the pandemic situation in 2020, the translation of the English version of the original instrument was done collaboratively by the members of the research team. Specifically, we used an iterative committee approach [55]. The team also included the abovementioned master’s students. The quality control role was assigned to the first author, who was closely familiar with the original study and with a broad context of the emerging literature on DCT. He did not participate in the translation iterations directly, and these iterations were facilitated by the second author. Apart from the clarity of translation, the first author also independently verified the final version of the instrument for appropriateness of cultural adaptation [56].

An example of lexical problems identified during the quality checks included the item PSE3, which was in the English version phrased as “If I were infected by the COVID-19 virus, my health would be significantly reduced.” As the word-for-word translation would result in a strange and not natural linguistic construction in the Czech language, the priority was eventually given to semantic similarity by translating the item as “Kdybych byl/a nakažen/a virem COVID-19, můj zdravotní stav by se významně zhoršil” (literally, “If I were infected by the COVID-19 virus, my health status would significantly worse”). Less lexical problems were identified in the remaining scales. The phrasal expression, “be on guard,” contained in item PBE3 was translated into Czech as “být ve střehu” (literally, on the alert). The Czech version of the instrument is available in Multimedia Appendix 1.

As this replication study closely followed the original methodology, including the survey instrument with no major changes except translation, a separate pilot study was not performed.

For the cross-cultural comparison, we used the 6D model of national culture by Hofstede et al [43]. This perspective allowed us to expand the *Discussion* section with cross-cultural aspects that may influence the different results between both nations. We focused on 3 dimensions of the 6D model by Hofstede et al [43] that differ notably between the Czech Republic and Belgium (Table 1): *indulgence versus restraint* (IVR; Δ=28), *uncertainty avoidance index* (UAI; Δ=20), *individualism versus collectivism* (IDV; Δ=17).

**Table 1:** A comparison of Hofstede dimension between the Czech Republic and Belgium.

Dimension	Czech Republic	Belgium	Difference (Belgium – Czech Republic)
Power distance	57	65	8
*Individualism vs collectivism* ^a^	58	75	*17*
Masculinity	57	54	−3
*Uncertainty avoidance*	74	94	*20*
Long-term orientation	70	82	12
*Indulgance vs restraint*	29	57	*28*

^a^Top 3 dimensions with the biggest difference are italicized.

Regarding the first differing dimension in our comparison (IVR), “indulgence stands for a society that allows relatively free gratification of basic and natural human desires related to enjoying life and having fun” [57]. In contrast, restrain, prevailing in Central and Eastern Europe, “stands for a society that controls gratification of needs and regulates it by means of strict social norms” [57]. Among others, traits such as cynicism and pessimism are ascribed to restrained societies.

The second highest ranking difference is in UAI or a “society’s tolerance for ambiguity” [57]. Belgium has one of the highest rankings in the world; this means that Belgian citizens prefer to avoid uncertainty, try to plan their future, and avoid ambiguous or unknown situations. Belgian citizens are more conservative and rigid and tend to make safe and more conservative decisions than Czech citizens. In contrast, the Czech Republic population tends to score slightly low, that is, they have great tolerance for uncertainty and risky situations.

Finally, IDV refers to “the degree to which people in a society are integrated into groups” [57]. In highly individualistic cultures, one is expected to speak up and realize their own desires. In such an environment, group consensus is not necessarily expected, and such a culture may be portrayed as a sum of individuals rather than a coherent group coexisting in shared harmony. Of note, the right of privacy is articulated explicitly in these societies [57]. In contrast, what is valued in collectivism cultures is “tradition, conformity, and benevolence.” Moreover, in more collectivism cultures it is reasonable to expect high tendencies toward prosocial behavior [58]. Belgium has higher IDV values than the Czech Republic. It means that Belgian citizens prioritize themselves and their family more than society and place great emphasis on their independence (eg, work autonomy) and individual opinions.

### Model and Data Analysis Procedure

In accordance with the original study [13], we used the HBM to guide our quantitative analysis. The HBM is a well-established theoretical tool in the domain of social cognition applied to public health problems [28]. Quantitative analysis was performed using Jamovi (version 2.2.5 [59]) equipped with the *semlj* module (version 0.7.0), which is based on *lavaan* [60]. Consistent with the original study, we first analyzed the demographic characteristics of the respondents. We have presented them as frequencies, percentages, means, and SDs. Following the original study, we relied on the fit indicators, including comparative fit index (CFI), root mean square error of approximation (RMSEA), and standardized root mean square residual (SRMR). In addition, we examined average variance extracted (AVE).

An optional free-text question (“Please elaborate your opinion on usefulness/uselessness of the eRouska application and/or describe your personal experience in a more detail”) concluded the questionnaire and was used for qualitative analysis. Available free-text answers to this question from the survey participants were subjected to hybrid thematic analysis. This is a relatively common methodological approach for theory-driven analysis of free-text answers in surveys [61]. Specifically, we used a combination of inductive and deductive approach. The approach was deductive in that the analysis was informed by the previous studies, providing the theory-derived thematic baseline. In that sense, the central themes such as perceived susceptibility, perceived severity, perceived benefits, perceived barriers, cues to action, and self-efficacy were adopted from the original study by Walrave et al [20]. Following the qualitative study by Tretiakov and Hunter [62], the predefined list of major themes was further expanded to cover additional important aspects. With this additional conceptual layer, we aimed to cover more concrete dimensions that were not explored in the original study. On the basis of the study by Tretiakov and Hunter [62], we expected the major category, *patterns of use*, to reflect real-world user experience and concrete use cases when working with the eRouska app. Similarly, by adding *social influence* and *need for collective action*, we aimed to cover peer influence and the societal dimension of contact tracing apps.

Qualitative data were imported into MAXQDA Plus 2020 (version 20.4.2; Verbi Software [63]). The first author first familiarized himself with the free-text data through their repetitive reading. Then, he coded the data in an inductive manner by creating new codes that emerged from the data under respective major themes. By means of constant comparison, the fit between the respective codes and central themes was checked and the possible discrepancies were solved in an iterative manner by moving the codes across the central themes.

## Results

### Quantitative Results

#### Overview

The survey was opened by 1438 people, of which 903 (62.79%) started answering and 635 (44.16%) completed the survey (635/903, 70.3% completion rate). After applying the filtering criteria (ie, aged between 18 and 29 y), 81.7% (519/635) of valid responses were obtained. The mean age of the respondents was 21.9 (SD 2.53) years. Slightly more responses (281/519, 54.1%) were from women than from men. Only a minority of respondents (45/519, 8.7%) perceived themselves as members of a vulnerable group owing to the existence of a serious health condition. Less than half (224/519, 43.2%) of the respondents were current users of the Czech contact tracing app, eRouska. Most respondents (441/519, 84.9%) stated that they had not contracted COVID-19 yet or were not aware of the disease. Demographic characteristics of the respondents are summarized in Table 2, and study variables are presented in Table 3.

**Table 2:** Characteristics of the study sample (N=519).

Characteristics			Participants, n (%)
**Gender**			
	Men	238 (45.9)	
	Women	281 (54.1)	
**Age group (y)**			
	18-24	442 (85.2)	
	25-29	77 (14.8)	
**Educational level obtained**			
	Elementary school	2 (0.4)	
	Grammar school with matriculation examination	321 (61.8)	
	Grammar school without matriculation examination	4 (0.8)	
	Higher vocational school	7 (1.3)	
	University: bachelor’s degree	159 (30.6)	
	University: master’s degree	25 (4.8)	
	University: doctoral degree	1 (0.2)	
**Vulnerable health conditions**			
	Yes	45 (8.7)	
	No	474 (91.3)	
**Current use of eRouska (eMask)**			
	Yes	224 (43.2)	
	No	289 (55.7)	
	Do not know	6 (1.2)	
**Contracted COVID-19 in the past**			
	Yes	78 (15)	
	No	278 (53.6)	
	Do not know	163 (31.4)	

**Table 3:** Study variables.

Constructs and items			Original study^a^, mean (SD)		Our study^b^, mean (SD)		Cronbach α	
**Behavioral intention (BI)**								.956
	BI1: I would be willing to use the COVID-19 app.	3.18 (1.41)		3.28 (1.38)				
	BI2: I plan to use the COVID-19 app.	3.08 (1.40)		3.01 (1.46)				
	BI3: I want to use the COVID-19 app in the future.	3.18 (1.41)		2.94 (1.39)				
**Perceived susceptibility (PSU)**								.503
	PSU1: I am at risk of being infected by the COVID-19 virus.	2.86 (0.95)		4.10 (0.79)				
	PSU2: It is likely that I would suffer from the COVID-19 virus.	3.40 (0.99)		3.35 (1.02)				
	PSU3: It is possible that I could be infected by the COVID-19 virus.	3.18 (1.07)		3.34 (1.13)				
**Perceived severity (PSE)**								.898
	PSE1: If I were infected by the COVID-19 virus, it would have important health consequences for me.	3.74 (1.02)		2.65 (0.97)				
	PSE2: If I were infected by the COVID-19 virus, my health would be severely affected.	3.70 (1.04)		2.49 (0.91)				
	PSE3: If I were infected by the COVID-19 virus, my health would be significantly reduced.	3.79 (1.01)		2.53 (0.92)				
**Perceived benefits (PBE)**								.867
	PBE1: The COVID-19 app will offer me the opportunity to contribute to better knowledge about the spread of the virus.	3.49 (1.17)		3.56 (1.10)				
	PBE2: With the COVID-19 app, I will collaborate to reduce the spread of the COVID-19 virus.	3.38 (1.23)		3.47 (1.09)				
	PBE3: Thanks to the COVID-19 app, I will be more on my guard when I have face-to-face contact.	3.36 (1.23)		2.86 (1.13)				
	PBE4: Thanks to the COVID-19 app, I will take more precautions not to spread the COVID-19 virus myself (eg, wash my hands, maintain distance from others [social distancing], limit my outside movements).	3.18 (1.26)		2.16 (0.99)				
	PBE5: By using the COVID-19 app, I will help public authorities to combat the COVID-19 virus.	3.45 (1.20)		3.46 (1.02)				
	PBE6: The COVID-19 app will allow me to protect myself from the COVID-19 virus.	3.37 (1.17)		2.37 (1.07)				
**Perceived barriers (PBA)**								.701
	PBA1: The COVID-19 app will reduce its users’ privacy.	3.69 (1.11)		2.98 (1.19)				
	PBA2: The COVID-19 app will create tensions between individuals who are infected by the COVID-19 virus and those who are not.	3.61 (1.09)		3.09 (1.12)				
**Cues to action (CTA)**								.525
	CTA1: Website of a newspaper, TV or radio station, or magazine.	4.14 (1.82)		3.09 (1.17)				
	CTA2: App of a newspaper, Tv or radio station, or magazine.	2.89 (2.03)		2.78 (1.39)				
	CTA3: News shared on social media (Facebook, YouTube, Twitter, Instagram, etc).	3.68 (1.87)		3.36 (1.29)				
	CTA4: News shared through messaging apps (personal messages through WhatsApp, Messenger, etc).	2.99 (1.95)		2.15 (1.19)				
	CTA5: Alerts through email and newsletters.	2.94 (1.81)		1.17 (0.53)				
**Self-efficacy (SE)**								.666
	SE1: I have the knowledge needed to use the COVID-19 app.	3.62 (1.23)		4.35 (0.94)				
	SE2: I have the necessary resources to use the COVID-19 app.	3.78 (1.21)		4.55 (0.85)				
	SE3: I can get help from others if I experience difficulties using the COVID-19 app.	3.71 (1.14)		3.85 (1.15)				

^a^The study by Walrave at al [20], conducted in Belgium in April 2020.

^b^This study, conducted in the Czech Republic in October 2020.

#### Measurement Model

On the basis of the fit indicators and especially RMSEA, our application of the original measurement model as designed by Walrave et al [20] resulted in a worse fit than in the original study. Our analysis yielded the following indicators: *χ*^2^_254_=810; *P*<.001; CFI=0.995; RMSEA=0.065, 90% CI 0.060-0.070; and SRMR=0.070. In contrast, the study by Walrave et al [20] reported *χ*^2^_254_=750.9; *P*<.001; CFI=0.976; RMSEA=0.036, 90% CI 0.033-0.039; and SRMR=0.034. To identify a possible cause, we performed an analysis, as described in the following section.

In our case, except for certain items in the perceived susceptibility and cues to action constructs, all factor loadings (fls) were significant and above the threshold of 0.4 [64]. The items that did not fulfill the criterion of having an fl with the stated minimal value were as follows: PSU1 (“I am at risk of being infected by the COVID-19 virus”; fl=0.321), PSU3 (“It is possible that I could be infected by the COVID-19 virus”; fl=0.231), CTA1 (“Website of a newspaper, TV or radio station, or magazine”; fl=0.36), and CTA5 (“Alerts through email and newsletters”; fl=0.2). Taking that into consideration, we then examined the AVEs for all the constructs. We found that the model showed unsatisfactory AVE values (ie, values below the recommended threshold of 0.5 [64]) with respect to 2 constructs: perceived susceptibility (AVE=0.37) and cues to action (AVE=0.26). These AVE values indicate that “more variance remains in the error of the items than in the variance explained by the (two) construct(s)” [64]. All the remaining AVE values in the measurement model were >0.59.

Owing to this unsatisfactory performance of the measurement model, we opted for a consideration of removing some of the indicators of perceived susceptibility and cues to action. The decision about whether an item should be removed was guided by the recommendation of Hinkin [65]. The suggestion articulated by Hinkin [65] is that the correlation coefficient value of 0.4 should be viewed as a reasonable minimal threshold for deciding whether to delete an item that is “producing error and unreliability.” Therefore, we examined interitem correlations for the first construct (perceived susceptibility). We found that PSU2 (“It is likely that I would suffer from the COVID-19 virus”) correlated at 0.38 and 0.18 with the remaining 2 items, PSU1 and PSU3, respectively. On that basis, we removed PSU2 from the perceived susceptibility scale. With that adjustment, we improved AVE of the scale to 0.521.

Regarding cues to action, the situation was less straightforward. The interitem correlations are summarized in Table 4. When considering those values as a starting point, it appeared that in the examined cohort, the original scale of Walrave et al [20] measured several different facets of cues to action. Although the sole value of the correlation coefficient seen in Table 4 might suggest removing the items CTA1 to CTA3, one should also consider the low loading of CTA5 and the fact that according to common logic, CTA5 might not be a fitting measurement item, considering the characteristics of the study cohort. We eventually decided to reduce the scale to CTA1 and CTA2 by removing CTA3 to CTA5. Although the coefficient for intercorrelation between CTA1 and CTA2 is below the suggested threshold and a similar statement can be made with reference to the loading of CTA1, the chosen suboptimal solution appears to be reasonably straightforward in terms of model interpretation. Nevertheless, the described adjustment improved AVE to only 0.38 and, in that sense, did not result in the value of AVE >0.5. This means that even with the modified form of the cues to action construct, more variance remains in the error of the items. This is a limitation that is further discussed in the *Discussion* section.

After these adjustments, there was an improvement in the model parameters (*χ*^2^_168_=407; *P*<.001; CFI=0.998; RMSEA=0.052, 90% CI 0.046-0.059; and SRMR=0.050). This means that, based on RMSEA itself, the model fit slightly exceeds the desired maximum value of 0.5. Consistent with the original study, we subsequently included 2 covariates (ie, gender and COVID-19 personal health risk). We refrained from including the remaining 2 covariates (ie, age and education) used in the original study. Arguably, owing to the homogenous character of our sample, including the latter covariates would have resulted in a nonconvergent model, as attested during our analysis. In contrast, we added 1 more covariate not used in the original study—whether the person is a user of the Czech contact tracing app, eRouska.

We found that existing health condition was significantly related to perceived severity (β=.47; *P*<.001). Gender was not related to any of the variables. Being an eRouska user was significantly related to perceived barriers in the inverse sense (β=–.31; *P*<.001). In addition, being an eRouska user was significantly related to behavioral intention (β=.56; *P*<.001).

**Table 4:** Interitem correlations for the cues to action (CTA) construct.

Items	CTA1	CTA2	CTA3	CTA4
CTA1	—^a^	—	—	—
CTA2	0.31	—	—	—
CTA3	0.18	0.24	—	—
CTA4	0.15	0.23	0.38	—
CTA5	0.20	0.20	0.11	0.47

^a^Not applicable (only lower triangular part displayed for better readability).

#### Structural Model

**Figure 1:**
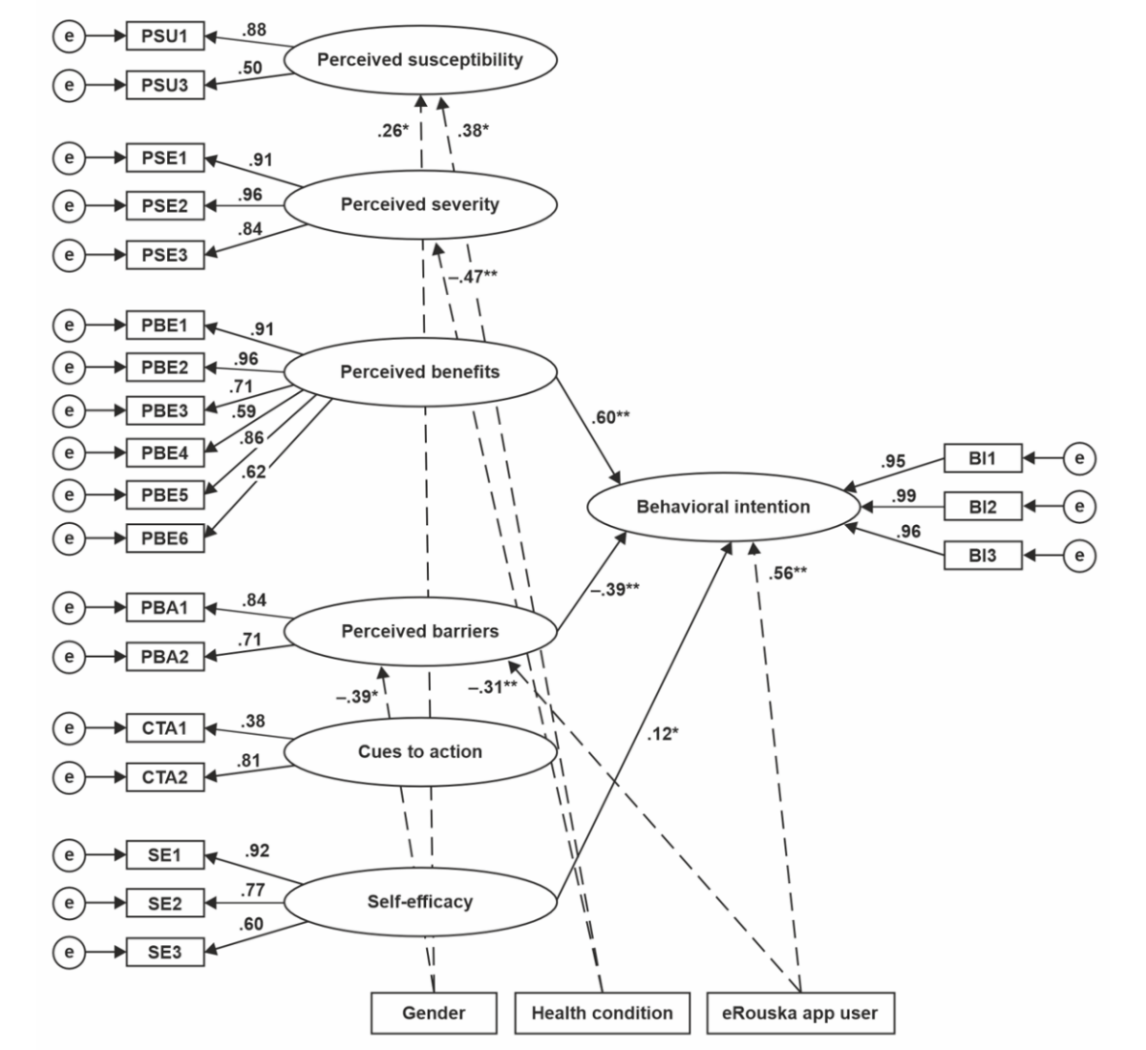
Structural model (the figure was created by the authors following the notation used in the original study). Nonsignificant paths are not included. Dashed lines refer to covariates. BI: behavioral intention; CTA: cues to action; PBA: perceived barriers; PBE: perceived benefits; PSE: perceived severity; PSU: perceived susceptibility; SE: self-efficacy. *P<.01 and **P<.001.

Figure 1 presents the results of the structural model. On the basis of the fit indicators, the adjusted model exhibits an acceptable fit (*χ*^2^_213_=455; *P*<.001; CFI=0.998; RMSEA=0.047, 90% CI 0.041-0.053; and SRMR=0.049). Judged solely from the values of RMSEA and SRMR, it is worse than that in the original study (*χ*^2^_350_=1070.46; *P*<.001; CFI=0.966; RMSEA=0.037, 90% CI 0.035-0.040; and SRMR=0.042). Consistent with Walrave et al [20], the most important predictor of intention was perceived benefits (β=.60; *P*<.001). Being the second most important predictor (inverse) of intention (β=–.39; *P*<.001), perceived barriers played a stronger role in our cohort than in the original study (reported as the third most important predictor). Self-efficacy scored with the third highest coefficient in our study instead of the second in the original study but did not achieve significance in the strict statistical sense (β=.12; *P*=.003). The remaining predictors were not statistically associated with intention.

### Qualitative Results

#### Overview

From the sample of 519 responses, we obtained 204 (39.3%) free-text answers to the optional question concluding the questionnaire. In summary, 49 unique codes and 354 total codes (ie, code instances) were created during the hybrid coding process. Illustratively, Table 5 lists the major themes and the frequencies of the associated code instances. We have reported the qualitative findings following the structure of the analytical categories introduced in the *Quantitative Results* section in a descendant order, based on their relative frequency in the free-text answers.

**Table 5:** Frequencies of the total codes (n=354) corresponding to major themes (n=6).

Major themes	Code instances, n (%)
Perceived barriers	166 (46.9)
Patterns of use^a^	55 (15.5)
Perceived benefits	48 (13.6)
Need for collective action^a^	48 (13.6)
Social influence^a^	12 (3.4)
Cues to action	6 (1.7)
Perceived susceptibility, Perceived severity, Self-efficacy	0 (0)
No explicit opinion or neutral opinion	19 (5.4)

^a^The major themes introduced by Tretiakov and Hunter [62].

#### Perceived Barriers

Of the analyzed free-text statements, so far, most were related to the barriers subjectively perceived by the respondents (*perceived barriers*). These barriers stemmed from a plethora of different concerns. Overall, 4 principal subthemes emerged from the data during the analysis: *unclear or missing benefit*, *psychological fears and concerns*, *inefficiency* (of eRouska), and *uselessness* (of eRouska). They are summarized in Table 6.

**Table 6:** Examples of free-text answers related to perceived barriers (the unique identifiers listed in brackets were generated by QuestionPro during data collection).

Subthemes	Sample comments
Inefficiency of eRouska	“[DCT exhibits] low efficiency, [stemming from] the low number of people involved.” [P^a^ 36352922] “It would make sense [to use the solution], if it was used literally by everyone. Not otherwise.” [P36728599] “I [repeatedly] receive the information about an encounter with an infected person 12-13 days following the encounter. I think that in such a case the application is pointless.” [P37106653] “I believe the application helps with [digital contact] tracing. Unfortunately, based on my experience, it [the process] takes quite a time. In my case, the contact with an infected person was indicated [only] after a week after the [supposed] contact. I waited 2 [additional] days for my code [to initiate the tracing of my own contacts].” [P37106731] “I know about some cases in my network, which were totally scamped [or even not contacted at all] by the people from the Public Health Service [original: “Hygiena”]. Given that even the Public Health Service is not of help, how eRouska can be?” [P36317552]
Uselessness	“Simply, I don’t feel a need to use the application, it appears pointless to me.” [P37102865] “A useless clue.” [P36417654] “The application is useful for those who meet an increased number of other people – especially when those are unknown to them – for an extended period of time.” [P37102497] “I think that in bigger cities or big shops it [the app] is useful. Personally, I don’t use it, because I live in a small town and don’t meet others often.” [P36312452]
Psychological fears and concerns	“...The data inserted to the application eRouska might be exploited and [subsequently] my location and movement will be watched.” [P36312320] “A tool for narking off people.” [P37104349] “According to me, an increasing [level of] control by the state, the EU [European Union] and other similar organizations is coming in [through the app].” [P37111473] “[The app] triggers panic in people; [for example] when he [!] is alerted by the app that he met a person positively tested, like on a tram. According to my opinion, it is not well-thought from the perspective of mental aspects...The fear is powerful, and we should never neglect that! From my view, I would rather not know that I met someone [infected]. Personally, I suffered from the illness,...having only minimal symptoms.” [P36315300] “I don’t mean to burden my mind with a fear about meeting people.” [P36626768]
Unclear or missing benefit	“As it appears to me, more important than having the app installed is hand washing, keeping the distance whenever possible, wearing a mask at public places with a higher concentration of people, and staying physically fit.” [P36312332] “Frankly, I have been disappointed by the app, as it relates to infected people and those people who have an increased probability of meeting the infection. For super-market shopkeepers, great. But for me there is no benefit.” [P36449110]

^a^P: Participant.

#### Patterns of Use

Under this theme, 3 subthemes were included, see Table 7. First, a large number of statements were related to specific *technical issues* with the app. This class of statements indicated that respondents would have been willing to use the app but were unable to do so. Second, a few problems directly related to individual *user experience* were mentioned. Third, some respondents reported specific patterns or *specific use case scenarios* that indicated certain different ways of interacting with the app than the developers arguably primarily intended.

The remaining themes were not analytically split into subthemes during the coding process. The reason was that either collected qualitative evidence did not provide an adequate level of insight and richness (the case of *perceived benefits*), was repetitive (the case of *social influence*) or had a low number of corresponding free-text answers (the case of *cues to action*). Therefore, we have presented the examples of free-text comments in textboxes instead of tables.

**Table 7:** Examples of free-text answers related to patterns of use.

Subtheme	Sample comments
Technical issues	“I would like to use the app, but it is not compatible with the older versions of iOS [iPhone Operating System].” [P^a^ 37105924] “I had it [the eRouska app] installed during the first pandemic wave for a time, but due to the batter drainage (my phone was literally on fire at times) I reconsidered my decision. I came to the conclusion that it would be better for the service life of the phone to deinstall it.” [P36315803]
User experience	“A significant disadvantage is that when I come home and turn it [the eRouska app] off, it does not announce a [risky] encounter until I turn it on [by bringing the app to the foreground].” [P37124213]
Specific use case scenarios	“I use eRouska solely as a source of [information about] the actual ‘numbers’ [of infection] and measures.” [P36724659]

^a^P: Participant.

#### Perceived Benefits

The frequency of explicitly mentioning the benefits was considerably lower than the negatives. Being not strictly against the concept, some respondents admitted a potentially positive impact, while still staying quite reserved. Some others were more enthusiastic, yet not explicitly articulating the concrete benefits that the app provides. We provide a summary in Textbox 1.

**Examples of free-text answers related to perceived benefits. P: Participant.**

- “...Perhaps really useful.” [P37103431]
- “It has indeed a sense for some.” [P37105597]
- “Overall, I consider it a beneficial and useful project.” [P36312313]
- “...A really good idea.” [P36736591]
- “I also appreciate the up-to-date information about the number of executed tests and the like [displayed] in the app.” [P37106930]

#### Need for Collective Action

Some respondents mentioned the reaction to the pandemic as a collective responsibility of the society. In contrast, some others set a clear boundary line between responsible behaviors in a broad sense and eRouska. In rare cases, our respondents explicitly expressed their lack of interest in the matter or even articulated an openly countersocial attitude. The first 2 examples in Textbox 2 demonstrate the former case, and the remaining 2 the latter case.

**Examples of free-text answers related to need for collective action (the latter examples should be interpreted as a “need for action in the inverse sense”, ie, a refusal to act). P: Participant.**

- “I don’t see the app as preventing the user getting infected, rather it is a tool of social responsibility in that it prevents the potential infection from [further] spreading.” [P36724996]
- “...I behave responsibly to prevent infecting myself and others, and that’s not something for what I need an app.” [P37102853]
- “I have never been interested, I have never downloaded it and I have never dealt [discussed] this [matter] with anybody.” [P36423262]
- “I am an egoist skunk, and I don’t have eRouska, as I don’t care if I get infected.” [P36417408]

#### Social Influence

The importance of acquiring information about eRouska from peers before installing it was highlighted by some respondents, using similar statements. A small number of respondents touched upon the problem of the (missing) communication strategy that would have promoted eRouska more. Textbox 3 provides illustratory evidence.

**Examples of free-text answers related to social influence. P: Participant.**

- The app...was not recommended to me a few times [by the people in my network], so I followed the advice of my peers [literally: neighborhood] and did not download it, nor am I considering doing so. [P36297193]
- Reportedly...eRouska 2.0 should be more followed through and also be less dependent on the initiative of the Public Health Service [original: “hygienická stanice”]. It’s hard to judge whether that’s really the case...However, based on what I heard, the notification about an enouncement with infected person is delayed for several days. [P36407888]
- According to my opinion, the mobile app eRouska is a very good idea. Unfortunately, there is little information [about the app available] within the public space. Often, people fear being watched...They fear their data will be exploited. Young people, in my view, don’t exhibit that level of anxiety as the older people. This [behavior] may be, for example, due to some influencers who have talked about eRouska and have explained how the app works. Unfortunately, this information don’t find their way to elderly people... [P36736591]

## Discussion

### Summary

In this study, we aimed to understand whether there was a difference in stimuli driving DCT adoption among Czech youth in contrast to the population of Belgium. From a theoretical perspective, we also wanted to confirm whether the HBM was an apt tool to support such an effort.

Regarding the first (policy-oriented) aim, we first reiterate the following fact. In the context of Europe, the decision to adopt DCT was mostly left to people. This was because many Western countries fully relied on balancing “privacy and public health” [9]. In that sense, efficacy of these apps must have been demonstrated to the public to convince them to start using the apps on a voluntary basis. Against this backdrop, involving the public in dialogue appears to be critical from today’s positions. Nevertheless, this was rarely followed during the pandemic times. DCT is a salient example of mobile health technology designed rapidly and without significant involvement of the key users [5]. Generally, such an approach to IT design is considered to be very problematic when one aims to introduce effective, consumer-friendly, and sustainable mobile health solutions. Following this reasoning, we wanted to learn from the perspectives of the youth Czech population. This was to offer ways toward strengthening the reportedly low adoption of the Czech contact tracing app during the pandemic in this cohort (and a similar app in a possible future pandemic).

Regarding the latter (theory-oriented) aim, we conducted a theory-driven replication of the original study by Walrave et al [20]. Broadly speaking, the advantage of theory-driven research such as the study by Walrave et al [20] is the gradual development of a coherent body of knowledge through repetitive theory-building and theory-testing cycles. As a form of established theory, the HBM has a long-standing tradition in the health care domain [38,39]. Despite this position, it has also received some criticism [66]. In that sense, it is important to recall that the model was created to explain general health behavior in the context of disease prevention and that it is a “cognition model, i.e. a model that emphasizes the way an individual provides a rationale for their behaviour without particular reference to a social context” [67], that is, the suitability of the model for the given problem should not be taken for granted.

In our case, we tested the original theory in a different cultural context. However, as many would argue, when one is testing an a priori defined theory, they might be at risk of forcing “preconceived ideas” on their research data [68]. This might result in missing important problems not yet elaborated in the existing theory. Being aware of the possible limitations of the HBM, we consequently opted for a brief qualitative verification, that is, we strived to triangulate the quantitative results with the available qualitative evidence of free-text nature, in a systematic manner [69]. We consider this additional analysis as being of illustrative nature only, owing to the nature and scope of the available qualitative evidence.

In the following sections, we have first discussed the quantitative evidence and then the qualitative evidence. Then, we have mentioned comparable national-level studies.

### Principal Quantitative Findings

In the quantitative part, we examined our data following the HBM, as done in the original study. This section follows the structure and sequence of the original study when discussing the results related to the individual HBM constructs. Overall, we confirmed support for the relationships posited by the original model. Specifically, in our cohort (N=519) of Czech youth aged between 18 and 29 years and knowledgeable about the local contact tracing app, 2 of 4 predictors (ie, perceived benefits and perceived barriers) were statistically significant and consistent with the original study. A predictor (self-efficacy) exhibited a trend toward significance (*P*=.003), playing arguably a similar role as in the original population. The remaining predictor from the original study (cues to action) was rejected as being insignificant in our cohort. To put the differing results obtained in Belgium and the Czech Republic into context, we used the cultural dimensions from the 6D model of national culture by Hofstede et al [43] presented in the *Methods* section.

According to the model, *perceived susceptibility* and *perceived severity* were not factors important for app uptake intention. This finding is consistent with that of the original study and the meta-analyses of additional studies [70,71], reporting that perceived susceptibility and severity were weakly predictive of health behaviors. Threat appraisal is linked to the complexity of the pandemic situation. As mentioned in the original study by Walrave et al [20], the perceived threat may be diluted when disease preventive behavior is complex or not well known. By staying at home and limiting contacts, some people might have lowered threat severity perceived by them.

These contextual aspects influencing people’s concerns are relevant also for our study, which covered only young people. Owing to this focus, further differences in particular aspects can be found between both studies, as shown in Table 3 (study variables). In contrast to the Belgian study examining a broad sample, young people in the Czech Republic were well aware that they are exposed to the risk of infection (PSU1). However, they seemingly did not believe that in case of their infection, COVID-19 would have a significant impact on their health (PSE1-PSE3). Arguably, this was owing to their youth and good physical health. In addition, this seems to be consistent with the cultural disposition of both nationalities (UAI dimension). The Belgian citizens emphasize safety more than the Czech citizens, who have great tolerance for uncertainty and risky situations.

Future studies of threat appraisal may focus on the older population, where the importance of both factors for app adoption can be expected to increase. This would be of great interest in connection with possible future pandemics. The coming older population, as “digital immigrants” [72] skilled to use the app yet fearing their lives more (given their aging), may exhibit a different pattern of behavior.

The significance of *cues to action* in relation to app uptake intention differed between our study and the original study. In the case of Belgium, cues to action was a significant factor with a less salient role (β=.13; *P*<.001) in app uptake intention. In our study, this was an insignificant factor for app uptake intention. An aspect to consider when interpreting the results is the differing time when both studies were conducted. The original study was conducted in the spring of 2020 (the invent of the pandemic in Europe), whereas our replication was conducted only in November 2020. It is reasonable to expect that the pandemic was seen as an enormous threat especially at the beginning, when little details about the disease features and real impacts on one’s health were known. At that point of time, assumably shocked people could be paying a lot of attention to various media channels. As the pandemic progressed, many people might become accustomed, temporarily accepting the situation as a new, temporary reality emerged during the pandemic times. Moreover, in stressful situations, many people tend to avoid information about the related condition, instead of actively consuming more of them [73]. In addition, in the Czech Republic, government information was frequently contradictory, and many people tended to believe that there existed no clear and consistent containment strategy in connection with the COVID-19 pandemic. This might have also heavily influenced the level of attention people paid to various media channels. Taken together, this could bring the feelings of resignation, cynicism, and pessimism, which is a more typical cultural trait for the Czech Republic than for Belgium (IVR dimension).

Of note, no DCT app was available in Belgium in the spring of 2020. However, some proposed solutions were being discussed in the public space. In contrast, in November 2020, the eRouska app had been available in the Czech Republic for 6 months already. Moreover, in the Czech Republic, the relatively low computer literacy of the Public Health Service’s representatives arguably played a considerable role. The low level of computer literacy seemed to result in a low pace when dealing with the population that is infected and when notifying their potential contacts. Put differently, the insignificance of cues to action in our study can be perhaps attributed to the contradictory information presented in the media (eg, growing numbers of cases vs organizational problems in the DCT system), arguably resulting in a personal conflict between the urge to help by installing the app versus the pragmaticism (cynicism) connected with such effort, appearing to make little difference owing to the mentioned factors anyway. In some populations (especially among young people who consume web-based media more), all these problems could possibly lead to information overload and anxiety, which then result in information avoidance [73].

In addition to the recommendation for further research in the original study (ie, to focus on how the media reported about the COVID-19 crisis), it would be appropriate to focus on information avoidance and misinterpretation in individual regions, mainly among people with low health literacy. The results of such studies [74,75] could show how to communicate complex topics to different social categories.

The role of *self-efficacy* in relation to app uptake intention was different between the Czech Republic (β=.12; *P*=.003) and Belgium (β=.25; *P*<.001). We point to differing values of items SE1 to SE3 in Table 3, which provide some clues. Overall, young Czech adults scored high in terms of reported self-efficacy aspects. Assumably, this difference is little surprising; our focus was on youth, who are considered to be digitally native and fluent with technology [72]. In the future, therefore, self-efficacy should be investigated especially in connection with high-aged citizens.

*Perceived barriers* were an important factor for DCT app uptake both for young Czech adults (β=–.31; *P*<.001) and for Belgian citizens (β=–.21; *P*<.001). Table 3 additionally shows that privacy concerns (PBA1) were higher in Belgian citizens than in young Czech adults. In that sense, one can use the concept of privacy to discuss the differing results of our study and the original one. Privacy-related perceptions seem to be linked to both generational and cultural characteristics of respondents [76,77]. First, our study focused on young respondents, who might, in general, have fewer concerns about privacy than the older population. Second, the reason for the different results may also stem from a cultural trait (UAI dimension). Belgian citizens place more emphasis on individual safety (including ensuring privacy) than Czech citizens. Therefore, the conclusions of the Belgian study draw attention to the need to explain privacy protection when launching and promoting a DCT app to its users. Other studies [78-81] also show the importance of maintaining privacy for users of COVID-19 tracing apps, taking into account trust in the national public health service system [82].

Further studies should focus on three topics: (1) fear of misuse of data or information by the service provider (eg, geolocation data), (2) constant anxiety from the app’s sudden notification about an encounter with a person who is infected (refer to the following sections), and (3) studying the app’s contribution and effectiveness in the broad context of the entire contact tracing system.

In both our study and the original study, the most important factor regarding app uptake intention was *perceived benefits*. However, in the case of the Czech Republic, the importance attributed to this factor was even higher (β=.60; *P*<.001) than in Belgium (β=.41; *P*<.001). This may again be related to the respondents’ age and cultural differences. Young people have a more positive attitude toward new technology, as they live in a “virtualized society” that is an integral part of their reality [72]. The group also called “digital natives” is more tech savvy, with more confidence when working with technology [41,42]. It follows that it is easy for them to understand how a particular technology works and what potential it may bring. In terms of cultural traits, Czech citizens are more collectivist than Belgian citizens (IDV dimension), which may imply a certain level of altruism [83]. Perceived prosocial benefits could have motivated some of the Czech citizens to install the app [24]. In terms of their technical skills, they might be fully aware of the necessity of increasing the number of app users among the general population to make the contact tracing mechanism work. Unfortunately, this initial enthusiasm might have been considerably eroded through time owing to additional factors. Again, these arguably included long reaction times of the workers of the Public Health Service, whose personal involvement in the process of contact tracing was necessary to notify contacts who are potentially infected through eRouska.

Further studies in this area may focus on incentive mechanisms in individualistic and collectivistic nations. The understanding of these mechanisms can help to emphasize the positive outcomes of DCT. Moreover, during a pandemic, it is desirable to clearly outline the benefits of using the app in the context of a complex antipandemic strategy.

### Outcomes of Brief Qualitative Verification of the HBM

The *Qualitative Results* section summarized the results of a qualitative validation of the HBM performed in the context of contact tracing apps. Notably, Table 5 presented the relative frequencies of major themes derived from the HBM. The results of the qualitative study complement the discussion about the quantitative results in the previous section and bring a broad view into the contextual background of the potential adoption of the app. On the basis of this additional analysis, we have provided several considerations for further application of the HBM in the domain of DCT in the following section.

First and foremost, the high frequency of the top category (perceived barriers) indicates that it could be worthy to re-examine the operationalization of the perceived barriers construct in terms of the diversity of the individual motives blocking the adoption. When designing the present form of the survey instrument, the authors of the original study seemingly assumed that the barriers would be primarily related to the privacy concerns and to the creation of “tensions between individuals who are infected by the COVID-19 virus and those who are not” [20]. Using our qualitative results, however, we have indicated that other subjective, cognition-driven perceptions about low efficiency of the technology (or its complete “uselessness”—a word used by a number of respondents) also could play an important role. In that sense, previous studies have shown significant polarization in many societies regarding the severity of the pandemic crisis and what measures are considered as appropriate reactions at the societal level [84]. In terms of qualitative results, this polarization can be confirmed in the context of the DCT technology implemented in the Czech Republic. We hold that a conceptual development of the perceived barriers construct could help with more precise capturing of the important nuances associated with citizens’ resistance toward DCT [31].

In contrast, we need to underscore the following aspect. The high ranking of various perceived barriers among the free-text answers might be owing to the cultural context in which this replication was conducted. As a case of more restrained cultures (IVR dimension), the Czech citizens appear to be quite vocal regarding various negative aspects of everyday lives. As a matter of fact, positive emotions tend to be pronounced in the Czech culture much less frequently. This particular cultural facet seems to repeatedly secure the Czech citizens top positions in popular rankings cross-culturally examining the trait of pessimism [44,85,86].

Second, the HBM appears not to be adequately equipped to capture psychological fears and concerns. We argue that these cognitive triggers might result in forming a specific type of perceived barrier [87]. To illustrate, some of our respondents had an attitude that can be colloquially summarized as “better not to know,” that is, they avoided contact tracing–related information by eluding “searching for (such a) potentially distressing information” [88]. Unfortunately, the operationalization of the HBM used in the original study was not able to account for such a set of attitudes. Importantly, within the body of knowledge of health care sciences and communication research, the already mentioned phenomena of *information avoidance* is not new [88]. Many relevant studies can be found in the areas such as research focusing on the quality of life of survivors of cancer [89] and cancer genetics [90]. Perhaps of more relevance to this study, this problem has also been addressed in connection to information-seeking behavior of citizens during the pandemics [73]. On the basis of the presented qualitative data, we consider accounting for the possible role of purposefully avoiding pandemic-related information by some citizens as essential for future studies conducted in this area.

Next, our respondents mentioned a large number of issues directly related to technology and user experience aspects. Although we consider this area as being of great importance for the designers and developers of similar apps, one needs to admit that the HBM is certainly not the best conceptual means for analyzing those issues. Simply stated, those issues do not align with the HBM’s psychological orientation. Moreover, those issues are mostly bound to the specific national context, as different countries pursued different strategies when building the digital infrastructure for DCT during the pandemic times. Therefore, we do not discuss this class of problems in detail in this section.

Finally, an additional area briefly highlighted by our study and confirmed by other studies dealing with contact tracing is social influence and the awareness of the “need for collective action” [62]. In the conceptualization put forward in this study, the former area would be covered by additional “cues to action” (a term widely used in HBM studies) obtained from informal social interactions (eg, from friends and family) rather than official media. The fact that we did not find the existing composite of cues to action as statistically significant in our replication may further explain the important role that informal social mechanisms seemingly played in our cohort.

In contrast to social influence, the need for collective action covered subthemes related to the desirability of prosocial behavior during the pandemics and taking individual responsibility, in a broad sense (eg, by wearing a mask). Again, the present conceptual apparatus of the HBM seems to be of very limited help at best. Studies of prosocial behaviors during the pandemics is an area that significantly expanded during the pandemics [30,91,92]. Referring to personality psychology literature, one can formulate the following assumption. There are individual personality factors that result in one’s strong perception about the benefit in taking a collective action for society as a whole during times of crisis [93]. Apart from the examination of the role of individual personality, mapping the role that national culture might play in prosocial behavior is an important yet extensive task [27].

Both of the previously listed deficiencies seem to call for reconsidering how these additional drivers, including peer influence and perceptions about the necessity for collective action, could be more accurately reflected in future studies using the HBM. Both peer influence and information avoidance can be incorporated into the HBM, for example, by including additional “modifying variables.” Such an approach was suggested by O’Dwyer et al [94]. In their case, they demonstrated certain conceptual limitations of the HBM by highlighting the power of peer communities in the context of sexual risk behavior.

In summary, our brief qualitative verification offers 3 important lessons to be considered when applying the HBM in future studies. First, it will be useful to extend the scope of the barriers expected to be perceived by citizens in connection to DCT by following recent studies. In addition, when studying the adoption processes of a pandemic-related technology, one should also consider a significant mass of people who reject any pandemic measures *principally*. Therefore, not all perceived barriers must have a rational foundation. Second, in connection to the previous aspect, human fears and concerns play an important part in human decision-making processes, and not all human decisions are made on a rational basis. The COVID-19 crisis has elucidated the need for studying the influence of these cognitive forces in the pandemic context [87]. Finally, it appears very problematic to entirely omit the role of informal social interactions and social media platforms by focusing solely on cues for action derived from mass media. The social media platforms and “word-of-mouth” derived from face-to-face interactions simply seem to play a nonnegligible role in the adoption processes related to DCT. Evidence for such a role can be found in various academic domains, including business and management [95].

### Comparison With Other National-Level Studies

Highlighting the importance of cross-cultural comparisons [40], our study suggests that although certain conclusions from similar studies may be shared across Europe and Western countries, there also seem to be important differences between the nations. In this section, we put our study in the broad COVID-19 research context, including other nation-level studies that theorize the mechanisms of DCT adoption. Table 8 presents the key results derived from other national-level studies that involved the HBM. Studies with considerably different predictors than ours were excluded. Overall, the identified studies emphasize perceived benefits, perceived barriers (privacy concerns), and self-efficacy (ability to use the app) as the main predictors of DCT uptake.

**Table 8:** Comparison of the national-level study results related to the adoption of a contact tracing app for coping with COVID-19. The search for studies using the Health Belief Model (HBM) was conducted through Scopus in May 2023.

Study	Respondent information	Country	Research constructs used	Main conclusions regarding the most important predictors
Walrave et al [20], 2020	1500 respondents aged 18-64 y	Belgium	HBM	The uptake of contact tracing apps could be enhanced by factors related to perceived benefits and self-efficacy in the HBM. Privacy concerns represent a perceived barrier for some potential users.
This study (replication of the study by Walrave et al [20], 2020)	519 respondents aged 18-29 y	Czech Republic	HBM	Perceived benefits and perceived barriers were confirmed as the main predictors of contact tracing app uptake. In addition to privacy concerns, the perceived low efficiency of the technology was also an important barrier.
Nguyen et al [96], 2023	219 respondents aged >18 y; respondents aged 18-29 y make up 60% of the study sample	Vietnam	HBM, second-order construct of privacy concerns, and second-order construct of factors mitigating privacy concerns	Perceived benefits were twice as large as privacy concerns (ie, perceived benefits offset privacy concerns). Individual collectivism was revealed as a mitigator of the trade-off dilemma (cultural aspect).
Harborth et al [97], 2023	1752 respondents aged >18 y; respondents aged 18-29 y make up 21% of the study sample	Germany	HBM (cues to action were split into intrinsic and extrinsic motivation constructs)	Adoption is positively influenced by the intrinsic and extrinsic motivation (cues to action) of individuals and negatively influenced by perceived technical barriers, privacy concerns, and low income.
Zhang and Vaghefi [98], 2022	171 US respondents and 203 UK respondents	The United States and the United Kingdom	HBM	Perceived benefits, self-efficacy, perceived severity, perceived susceptibility, and cues to action positively predicted the continued use intentions of contact tracing app, whereas perceived barriers reduced them.
van Der Waal et al [99], 2022	1865 respondents aged >18 y	The Netherlands	HBM and Unified Theory of Acceptance and Use of Technology	Self-efficacy (most important), perceived barriers, and perceived benefits were associated with contact tracing app adoption.
Xie et al [100], 2021	255 respondents aged >18 y	Ireland	HBM, Privacy Segmentation Index, and Privacy Attitude Questionnaire	Perceived barriers (privacy attitude), cues to action (familiarity with the app and its role), and perceived benefits are the main factors influencing adoption.

Before listing the main limitations of our study, we would like to add a note regarding the role of the context in which the eRouska app was operated until October 1, 2021. On that day, the system was decommissioned. The full story of the eRouska app’s failure in the Czech Republic during the COVID-19 pandemic is yet to be told elsewhere. Despite that, we hold that this study supports the view that eRouska became a victim of 2 key factors only loosely conceptualized in the HBM: the government’s unprofessional media communication and low efficiency of the “people component” (ie, the Public Health Service) in the contact tracing mechanisms. The latter apparently stemmed from the low digital literacy featuring in certain Czech medical fields, including public health, before the pandemic [101]. This argument illustrates that relying purely on the quantitative evidence embodied by the HBM variables might be tricky. In that sense, we support the view of many perceptive researchers who argue that “Context is king!” [102] and that behavior-focused “theories in the social [as well as health] sciences are implicitly limited by cultural or contextual circumstances” [102].

### Limitations

Apart from the generic limitation mentioned previously, this study exhibits a number of more specific limitations. First and foremost, similar to the original study, we used a convenience sample. This comes at a price, and the presented findings cannot be generalized. We also focused on a narrower population than the original study; therefore, it is not possible to draw strong conclusions by directly comparing the results, given the characteristics of both samples. Moreover, the quantitative analysis performed in this replication showed that the proposed measurement model, based on the fit indicators, does not exhibit a particularly good fit when applied in the given setting. However, this should not be treated as a major threat of this replication study but rather as an impulse for a future development of the model. We argue that falsifiability of existing theories and models is one of the most crucial attributes of scientific inquiry. To assist in those efforts, we provided qualitative evidence that supports the finding that the model might be of problematic application in the cohort of young adults.

Regarding the qualitative results, one should acknowledge its supplementary role in this research project. In terms of its breadth and depth, one cannot expect that our qualitative data, which originated in a single free-text answer, could provide the insights comparable with a full-fledged qualitative study. However, we believe that the qualitative analysis conducted in this research project can illustrate the participants’ reasoning beyond the deployed quantitative scales. In other words, it is reasonable to expect that the free-text answers captured the “very first thing” many respondents had on their mind in the context of DCT. Overall, in similar types of research projects, it is always desirable to triangulate the qualitative data by using a more comprehensive qualitative method as a next step.

### Conclusions

In this study, we replicated the analytical approach of Walrave et al [20] by using the HBM when examining the predictors of DCT adoption. Although we found that the present model exhibited a less optimal fit than in the original study, it is possible to sum up the key findings as follows. In our cohort of young Czech adults aged between 18 and 29 years, we confirmed that perceived benefits and perceived barriers were the main, statistically significant predictors of DCT uptake. Although in the original study, self-efficacy also proved to be a predictor, in our study, this construct showed only a trend toward statistical significance. Taken together, we found considerable differences between the weights of predictors defining the structural models in our study and the original one. More importantly, when examining the measurement model in detail, we found that perceived severity and cues to action, as operationalized in the original study, exhibited insufficient content and convergent validity in our context. Future studies should therefore focus on reconceptualizing both constructs. It is our hope that the presented qualitative findings may be of help in such an effort.

In conclusion, we have argued together with other researchers [2] that cumulative evidence describing DCT adoption at the national level in individual countries may help local policy makers to improve crisis management strategies and to get ready for future pandemic threats. In the postpandemic times, governments should not be circumvented by possible future pandemic crises. They should prepare a complex and actionable portfolio, including informal, people-oriented strategies; formal organizational tactics and regulations; and new technologies, and have it ready at hand [2]. Part of this effort includes design, implementation, and operation of effective contact tracing systems. In the event of a future pandemic, developers of DCT apps should adhere to both generic and local (ie, derived from a particular cultural context) recommendations. The evidence provided by our study allows to do so with respect to the unique cultural context of the Czech Republic, and more broadly, Central Europe.

## Appendix

Multimedia Appendix 1 The Czech version of the instrument.

## Abbreviations

AVE: average variance extracted
CFI: comparative fit index
DCT: digital contact tracing
fl: factor loading
HBM: Health Belief Model
IDV: individualism versus collectivism
IVR: indulgence versus restraint
RMSEA: root mean square error of approximation
SRMR: standardized root mean square residual
UAI: uncertainty avoidance index
