# Supplementary material for: Adoption of a COVID-19 Contact Tracing App by Czech Youth: Cross-Cultural Replication Study
Source: JMIR Hum Factors. 2023 Nov 16;10:e45481. doi: 10.2196/45481 (PMC10655852; doi:10.2196/45481)
Supplement: Multimedia Appendix 1 [file humanfactors_v10i1e45481_app1.docx]

On-line appendix A – The Czech version of the instrument

**Behavioral intention**

BI1. Jsem otevřený/á používání aplikace eRouška

BI2. Aplikaci eRouška plánuji používat

BI3. Chci používat aplikaci eRouška v budoucnu

**Perceived susceptability**

PSU1. Existuje riziko, že se nakazím virem COVID-19

PSU2. Je pravděpodobné, že nákazu virem COVID-19 bych negativně pocítil/a na svém zdraví

PSU3. Je možné, že bych virem COVID-19 mohl/a být nakažen/a

**Perceived severity**

PSE1. Kdybych byl/a nakažen/a virem COVID-19, mělo by to pro mě výrazné zdravotní dopady

PSE2. Kdybych byl/a nakažen/a virem COVID-19, těžce by to dopadlo na moje zdraví

PSE3. Kdybych byl/a nakažen/a virem COVID-19, můj zdravotní stav by se významně zhoršil

**Perceived benefits**

PBE1. Aplikace eRouška představuje možnost, jak mohu osobně přispět k lepšímu trasování (sledování) šíření nemoci COVID-19

PBE2. Díky aplikaci eRouška se mohu zapojit do společného úsilí s cílem omezit šíření viru COVID-19

PBE3. Aplikace eRouška mi umožní být více „ve střehu“ při osobním kontaktu

PBE4. Díky aplikaci eRouška se mohu chovat zodpovědněji (např. umýt si ruce, udržovat odstup od ostatních [sociální distancování], omezit svůj pohyb venku)

PBE5. Používáním aplikace eRouška mohu pomoci veřejným orgánům s bojem proti viru COVID-19

PBE6. Aplikace eRouška mi může pomoci chránit se před nákazou COVID-19

**Perceived barriers**

PBA1. Aplikace eRouška má negativní vliv na soukromí uživatelů

PBA2. Aplikace eRouška může vytvořit napětí mezi nakaženými a nenakaženými osobami

**Cues to action**

CTA1. Zpravodajský portál, televize, rádio či časopis

CTA2. Mobilní či jiná aplikace zpravodajského portálu, televize, rádia či časopisu

CTA3. Zprávy sdílené na sociálních sítích (Facebook, YouTube, Twitter, Instagram aj.)

CTA4. Zprávy sdílené přes chatovací aplikace (osobní zprávy přes aplikace jako jsou WhatsApp, Messenger, Telegram aj.)

CTA5. Upozornění zaslaná E-mailem (včetně newsletterů)

**Self-efficacy**

SE1. Mám znalosti a dovednosti nutné k používání aplikace eRouška

SE2. Vlastním zařízení vhodné pro instalaci aplikace eRouška

SE3. Vím o někom, kdo mi může pomoci s aplikací eRouška, pokud bych narazil/a na problémy
